# Altered Gray Matter Volume and White Matter Integrity in College Students with Mobile Phone Dependence

**DOI:** 10.3389/fpsyg.2016.00597

**Published:** 2016-05-04

**Authors:** Yongming Wang, Zhiling Zou, Hongwen Song, Xiaodan Xu, Huijun Wang, Federico d’Oleire Uquillas, Xiting Huang

**Affiliations:** ^1^School of Psychology, Southwest UniversityChongqing, China; ^2^Department of Neurology, Massachusetts General Hospital, Harvard Medical SchoolBoston, MA, USA

**Keywords:** mobile phone dependence, Mobile Phone Addiction Index scale, fMRI, gray matter volume, fractional anisotropy, axial diffusivity, impulsivity

## Abstract

Mobile phone dependence (MPD) is a behavioral addiction that has become an increasing public mental health issue. While previous research has explored some of the factors that may predict MPD, the underlying neural mechanisms of MPD have not been investigated yet. The current study aimed to explore the microstructural variations associated with MPD as measured with functional Magnetic Resonance Imaging (fMRI). Gray matter volume (GMV) and white matter (WM) integrity [four indices: fractional anisotropy (FA); mean diffusivity (MD); axial diffusivity (AD); and radial diffusivity (RD)] were calculated via voxel-based morphometry (VBM) and tract-based spatial statistics (TBSS) analysis, respectively. Sixty-eight college students (42 female) were enrolled and separated into two groups [MPD group, *N* = 34; control group (CG), *N* = 34] based on Mobile Phone Addiction Index (MPAI) scale score. Trait impulsivity was also measured using the Barratt Impulsiveness Scale (BIS-11). In light of underlying trait impulsivity, results revealed decreased GMV in the MPD group relative to controls in regions such as the right superior frontal gyrus (sFG), right inferior frontal gyrus (iFG), and bilateral thalamus (Thal). In the MPD group, GMV in the above mentioned regions was negatively correlated with scores on the MPAI. Results also showed significantly less FA and AD measures of WM integrity in the MPD group relative to controls in bilateral hippocampal cingulum bundle fibers (CgH). Additionally, in the MPD group, FA of the CgH was also negatively correlated with scores on the MPAI. These findings provide the first morphological evidence of altered brain structure with mobile phone overuse, and may help to better understand the neural mechanisms of MPD in relation to other behavioral and substance addiction disorders.

## Introduction

As reported by eMarketer.com, the number of universal smartphone subscribers will reach 2,380 million in 2017, 672.1 million of which will be Chinese subscribers. Smartphones have many attractive characteristics that help foment its prevalent use in modern life, particularly for young adults. It is an inexhaustible source of fun and relaxation, a highly effective instrument for establishing and maintaining interpersonal relationships, and it is a convenient method for avoiding unpleasant mood states and ‘killing’ time (Choliz, 2010).

Increasingly, individuals perceive their world via this sophisticated, multifunctional, new ‘organ.’ However, more and more young adults are using smartphones in an out of control fashion. In recent years, physiological, social, behavioral, and even affective problems have been associated with extensive, uncontrolled, and excessive use of mobile devices, drawing a lot of attention to the potential negative effects of mobile phone overuse (Roberts et al., 2015).

Mobile phone overuse, as a disorder, can be considered a behavioral addiction (Billieux, 2012). The traditional general framework for addiction is based on a medical model that refers to bodily and psychological dependence from the consumption of substances such as tobacco, alcohol, or other drugs (McMillan et al., 2001). However, researchers have argued that addiction should be expanded to include pathological behavioral patterns that are analogous to substance dependence, and they have collectively referred to these as ‘behavioral addictions’ (Lemon, 2002). Behavioral addiction thus refers to behaviors, besides psychoactive substance ingestion, that produce short-term feelings of reward and engender further persistent behavior despite knowledge of adverse consequences. These behaviors include pathological gambling, skin picking, kleptomania, compulsive buying, and compulsive sexual behavior, to name a few (Grant et al., 2010). Behavioral addiction resembles substance addiction in many domains, including natural history, phenomenology (Roberts et al., 2015), tolerance (Leung, 2008), overlapping genetic contributions (Billieux, 2012), neurobiological mechanisms (Billieux et al., 2015a), comorbidity, response to treatment (Billieux et al., 2015b), and the common core feature of diminished control (Walther et al., 2012). With the advent and growing ubiquitous use of technology such as television, computer gaming, and the Internet, a new subclass of behavioral addiction that is non-chemical in nature, technological addiction, has been characterized as the problematic excessive use of technology involving human–machine interaction (Griffiths, 1996).

Mobile phone dependence (MPD), a subset of behavioral or technological addiction, shares lots of common features with other addiction disorders (Bianchi and Phillips, 2005; Billieux, 2012). These common characteristics are summarized by ‘Brown’s behavioral addictions criteria’ (Brown, 1993), and include: cognitive salience, conflict with other persons or activities, euphoria or relief, tolerance or loss of control over the behavior, withdrawal, relapse, and reinstatement (Martinotti et al., 2011). Overall, MPD has been depicted as the excessive and uncontrolled use of a mobile phone to the extent that it affects an individual’s real-life. For example, reminiscent of dependence and withdrawal, someone with MPD may feel uncomfortable and irritated in the absence of their phone, including feeling a physical and psychological void among other classical symptoms of withdrawal (Ling and Pedersen, 2006).

For many MPD individuals, a mobile phone can be so fascinating that it can dominate their life and interests (Chóliz, 2012). In fact, research has indicated that MPD is related to psychological distress, emotional instability, materialism (Beranuy et al., 2009), approval motivation (Takao et al., 2009), leisure boredom, sensation seeking (Leung, 2008), impulsiveness (Billieux et al., 2007, 2008), and risky behavior such as unprotected sex, illicit drug use, alcohol consumption, suspensions from school, and criminal activity (Yang et al., 2010). Furthermore, negative associations have been found between MPD and healthy introversion and conscientiousness (Roberts et al., 2015), working memory (Billieux et al., 2008), executive function (Billieux, 2012), self-control and self-monitoring (Takao et al., 2009), and even self-esteem (Yang et al., 2010). MPD may also cause lowered cognitive control, elevated reward seeking behaviors, increased tolerance to phone exposure, and impaired psychological health as well as lead to reduced work performance and even academic failure (Billieux et al., 2015a), similarly to other addiction disorders.

Importantly, trait impulsivity has been shown to play a key role in many kinds of dependence disorders including drug addiction (Moreno-López et al., 2012), gambling problems (Joutsa et al., 2011; Bickel et al., 2012), online game addiction (Han et al., 2012b), and even internet addiction (Cao et al., 2007; Lin et al., 2012). Thus, in the present study, we considered measuring possible underlying impulsivity in MPD as well.

Though some potential factors that may be related to MPD have been identified in previous studies, no body of research has yet been performed that investigates the underlying neural mechanisms or possible brain morphological alterations that are present in MPD individuals. There is tremendous promise for using Magnetic Resonance Imaging (MRI) to elucidate MPD’s neural mechanisms (Yuan et al., 2011), and results may help the development of behavioral interventions or pharmacological treatments for this and other types of addiction in the near future (Hanlon and Canterberry, 2012). Thus, in the present study, we aimed to explore measures of brain morphology with MRI in individuals with MPD, particularly in young adult college students due to the increasing role that mobile phones play in this population.

Hypothesized common underlying neural patterns between MPD and other addiction disorders lend to the idea that by better understanding the mechanisms behind MPD, other types of addiction may be elucidated too (Billieux et al., 2015a). In a study of internet addiction in young adults, Zhou et al. (2011) found that compared with a healthy control group, internet-addicted young adults had lower gray matter density in the left anterior cingulate cortex, left posterior cingulate cortex, left insula, and left lingual gyrus. In a similar study looking at online game addiction in young adults, Weng et al. (2013) found gray matter atrophy in the right orbitofrontal cortex (OFC), bilateral insula, and right supplementary motor area, as well as reduced fractional anisotropy (FA) in the right genu of the corpus callosum, bilateral frontal lobe white matter (WM), and right external capsule in online game addicted-individuals. In research concerned with pathological gambling, there was higher gray matter volume (GMV) in the ventral striatum and right prefrontal cortex (Koehler et al., 2013), widespread lower FA, and higher mean diffusivity (MD) in the corpus callosum, the cingulum, the superior longitudinal fascicle, the inferior fronto-occipital fascicle, the anterior limb of the internal capsule, the anterior thalamic radiation, the inferior longitudinal fascicle, and the uncinate/inferior fronto-occipital fascicle in the group of pathological gambling patients (Joutsa et al., 2011). These reported regions are related to inhibitory control, reward processing, and impulsivity (Romero et al., 2010; Li et al., 2015). MPD may in theory also involve damage to some of these regions too (Hanlon and Canterberry, 2012), and exploring the similarities and differences among various addictions could deepen our understanding of the neural mechanisms of MPD behaviors and may help the development of specific interventions for it.

Many automated and objective MRI methods have been used to characterize healthy brain structural patterns, including T1-weighted structural imaging and diffusion tensor imaging (DTI). In the former, GMV can be inspected and further calculated by voxel-based morphometry (VBM) analysis. Based on the review above, we hypothesized decreased GMV in frontal lobe areas and thalamus in the MPD group relative to controls. We also supposed that the MPD group would be associated with impairment of WM fibers linked to emotional processing, executive attention, decision-making, and cognitive control. Four fiber integrity surrogates including FA, MD, axial diffusivity (AD), and radial diffusivity (RD), calculated via tract-based spatial statistics (TBSS) analysis (Yeh et al., 2009), are sensitive to the diffusion of water molecules in the brain (Basser et al., 1994), and are accurate qualified biomarkers for assessing the effectiveness of WM (Hasan et al., 2011).

In the present study, both GMV and WM integrity were explored using these measures in order to reveal potential discrepancies that may be present in young adults with MPD, with the potential to better understand its possible neural mechanisms.

## Materials and Methods

### Ethics Statement

This research was approved by the Ethics Committee of Southwest University, and written informed consent was obtained from every participant. All participants were over 18 years of age, and were notified that their participation was completely voluntary and that they had the ability to secede at anytime.

### Participants

Three hundred college students were recruited from Southwest University (SWU, Chongqing, China) by pamphlet and Internet advertisement. They were required to complete the Mobile Phone Addiction Index (MPAI) scale in which a score above 51 classified them as mobile phone dependent (MPD). This stratification led to an MPD group of 34 individuals (21 female, range: 18–27 years old). In order to match the MPD group, 34 non-MPD students (21 female, range: 18–27 years old) were randomly selected as the control group (CG). There were no significant differences in personal monthly expenses, age, gender, or years of education between groups (see **Table 1**).

**Table 1:** Economic status, demographics, MPAI and BIS scores of participants.

	MPD (*n* = 34)	CG (*n* = 34)	*t*	*p*
Monthly expenses (yuan)	954 (251)	928 (257)	0.43	0.67
Family income (yuan/months)	6265 (3028)	6559 (3228)	-0.39	0.70
Age (years)	21.60 (2.10)	21.73 (1.94)	-0.28	0.78
Years of education (years)	15.15 (1.37)	15.21 (1.37)	-0.18	0.86
Age of owning first phone	16.79 (2.41)	16.85 (2.22)	-0.10	0.92
Minutes of daily call-making	45.15 (30.34)	32.94 (16.93)	2.05	<0.05
Minutes of daily phone use	256.05 (100.95)	208.24 (90.40)	2.06	<0.05
MPAI scores	57.21 (5.98)	39.41 (6.45)	11.80	<0.01
BIS scores	47.50 (17.04)	37.60 (12.04)	2.77	<0.01

*MPD, mobile phone dependency; CG, control group; MPAI, Mobile Phone Addiction Index; BIS, Barratt Impulsiveness Scale; Values presented are means (standard deviation).*

Furthermore, all participants had no neurological disorders, history of psychiatric disorders, or metal parts, tattoos, or irremovable piercings on their body. They all had normal or corrected to normal vision, were right-handed, and were native Chinese speakers.

### Questionnaire Evaluation

The MPAI scale (Leung, 2008), which consists of 17 items, was used to estimate the degree of MPD. A five-point Likert scale was used: 1 = ‘not at all,’ 2 = ‘rarely,’ 3 = ‘occasionally,’ 4 = ‘often,’ 5 = ‘always.’ The total score ranges from 17 to 85 in which according to a median split, 51 or more is considered indicative of phone dependence (Martinotti et al., 2011). The reliability of the scale as indicated by Cronbach’s alpha is remarkably high at 0.90 (Leung, 2008).

The Barratt Impulsiveness Scale (BIS-11) (Patton et al., 1995) was administered to both groups to measure trait impulsivity. The BIS is made up of 30 items using a five-point Likert scale, where the higher the score, the stronger the impulsivity. The internal consistency reliability and retest reliability are 0.89, and 0.91, respectively (Li et al., 2011).

### Scanning Acquisition

All imaging data were acquired using a 3T Siemens scanner (Siemens Medical, Erlangen, Germany) at the Brain Imaging Research Center of Southwest University. High-resolution T1-weighted anatomical images were obtained with a magnetization prepared rapid gradient echo (MPRAGE) sequence [repetition time (TR) = 1900 ms, echo time (TE) = 2.52 ms, field of view (FOV) = 256 mm, flip angle = 90°, in-plane matrix resolution = 256 × 256, slice thickness = 1 mm, slices = 176, voxel size = 1 mm × 1 mm × 1 mm]. 12-direction diffusion tensor images (DTI) were collected with a twice refocused spin echo pulse sequence, TR = 6000 ms, TE = 89 ms, FOV = 240 mm, matrix resolution = 128 × 128, slice thickness = 3 mm, slices = 45, *b*-value = 1000s/mm^2^.

### Structural Imaging Data Analysis

Each subject’s structural imaging data was analyzed by Statistical Parametric Mapping software (SPM8^1^) in MATLAB R2014a (MathWorks Inc., Natick, MA, USA), and were first displayed to check for artifacts and gross anatomical abnormalities. Scans were manually co-registered and realigned to anterior commissure-posterior commissure line, then segmented into gray matter (GM), WM and cerebrospinal fluid (CSF) (Yin et al., 2013). Finally, the results were registered, normalized, and modulated using the Diffeomorphic Anatomical Registration Through Exponentiated Lie algebra (DARTEL) toolbox, which uses a more sophisticated registration algorithm to achieve more accurate inter-subject registration. Its performance on non-linear registration algorithms is better than that of other similar toolboxes.

After these preprocessing steps, the study-specific brain template was created from all subject images (i.e., mean image), and the image intensity of each voxel was modulated by the Jacobian determinants to facilitate the determination of regional differences in the absolute amount of GM. The registered images were then transformed to Montreal Neurological Institute (MNI) space, and finally, the normalized and modulated images were smoothed with a 10 mm full-width at half-maximum (FWHM) Gaussian kernel to increase signal-to-noise ratio.

The difference in GMV between the MPD and CG group was assessed by two-sample *t*-test using SPM8 in which total GM volume as well as BIS scores were added for covariance, as trait impulsivity may be a confounding factor. Absolute voxel signal intensity threshold masking was set to 0.2 to minimize GM boundary effects (Duan et al., 2012). Significance levels were set at *p* < 0.01 with Alpha-Sim correction, calculated using DPABI software^2^ (Chao-Gan and Yu-Feng, 2010). The resultant images were visualized with BrainNet Viewer (Xia et al., 2013).

In order to affirm differences associated with MPD relative to controls, the notably different GMV clusters between the two groups were selected as regions of interest (ROI), and the GMV values of these ROIs within the MPD group were then extracted by REST^3^, and entered into a Pearson correlation analysis with MPAI scores with significance set at *p* < 0.05 (Song et al., 2011).

### Diffusion Tensor Imaging Data Analysis

The diffusion-weighted imaging data were processed in the following way using the pipeline tool, PANDA^4^: estimation of the brain mask using the b0 image without diffusion weighting, cropping of the non-brain space in the raw images at 0.25 [fractional intensity threshold (0→1), where smaller values give larger brain outline estimates], correction of the eddy-current induced distortion and head motion during scanning by registering the diffusion weighted images (DWI) to the b0 image with an affine transformation, non-linear registration of all individual FA images in native space to the FA template in the Montreal Neurological Institute (MNI) standard space, warping of transformations used to resample the images of the diffusion metrics with 1 mm × 1 mm × 1 mm spatial resolution, execution of the TBSS procedure where all subjects’ skeletons were created and every subregion’s average FA, MD, λ_1_ and λ_23_ value of the subjects’ skeletonized WM were calculated according to the Johns Hopkins stereotaxic WM atlas – which comprises 50 core regions (Mori et al., 2008) – for further atlas-based count through multi-factor analysis of variance between the two groups while regressing out BIS scores. Finally, a Pearson correlation analysis was performed within the MPD group between the values from significantly different regions and MPAI scores with significance set at *p* < 0.05 (Cui et al., 2013). This method’s statistical power was tested reliably (Oishi et al., 2009; Faria et al., 2010). The outcome was presented with FSLView using the FSL toolbox (FSL 5.0.0^5^), (Smith et al., 2006).

## Results

### Questionnaire Performance

The MPD group had significantly higher scores on the MPAI than the CG. They also spent significantly more time on their mobile phone (see **Table 1**). As expected, BIS scores were significantly higher in the MPD group as well, showing that MPD individuals had higher trait impulsivity relative to controls.

### Gray Matter Volume Difference between Groups

**FIGURE 1:**
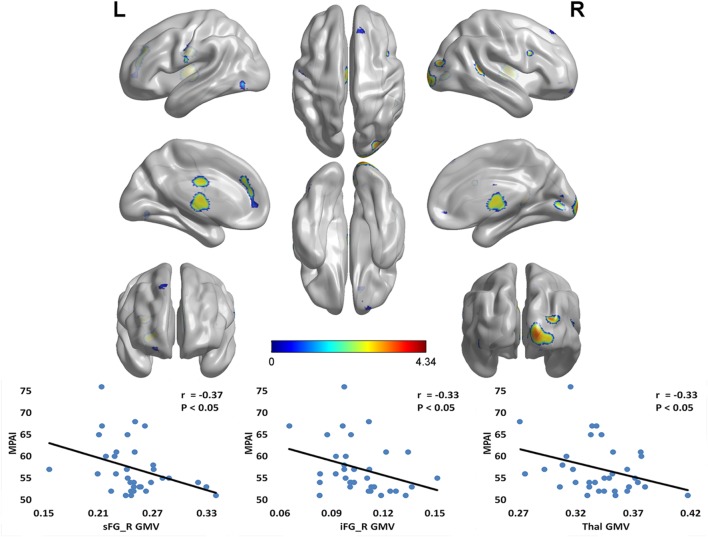
**Notable GMV differences between MPD group and control group (CG > MPD), and the negative correlation between GMV and MPAI scores within MPD group.** Compared with the control group, the MPD group had remarkably decreased GMV in the right sFG, right iFG, bilateral mFG, right mOG, left ACC, and bilateral Thal. Furthermore, within the MPD group, the GMV of the right sFG, right iFG and Thal was negatively correlated with MPAI scores. sFG, superior frontal gyrus; iFG, inferior frontal gyrus; mFG, medial frontal gyrus; mOG, middle occipital gyrus; ACC, anterior cingulate cortex; Thal, thalamus; MPD, mobile phone dependency; CG, control group; MPAI, Mobile Phone Addiction Index; GMV, gray matter volume. L, left; R, right.

Compared with the CG, the MPD group had remarkably decreased GMV in the right superior frontal gyrus (sFG), right inferior frontal gyrus (iFG), bilateral medial frontal gyrus (mFG), right middle occipital gyrus (mOG), left anterior cingulate cortex (ACC), and bilateral thalamus (Thal) (see **Table 2**). Furthermore, within the MPD group, GMV of the right sFG, right iFG and Thal was negatively correlated with MPAI scores (see **Figure 1**).

**Table 2:** Notable gray matter volume (GMV) differences between mobile phone dependent (MPD) group and control group (MPD group < control group).

Region	Peak MNI Coordinates			Voxels	*t*
	*X*	*Y*	*Z*		
sFG_R	16.5	42.0	54.0	163	3.31
iFG_R	60.0	25.5	9.0	153	3.11
mFG_L	-10.5	37.5	28.5	288	2.94
mFG_R	13.5	43.5	-9.0	89	2.83
mOG_R	31.5	-88.5	18.0	407	3.42
ACC_L	-13.5	46.5	10.5	115	2.95
Thal (L and R)	-1.5	-12.0	9.0	291	2.85

*sFG, superior frontal gyrus; iFG, inferior frontal gyrus; mFG, medial frontal gyrus; mOG, middle occipital gyrus; ACC, anterior cingulate cortex; Thal, thalamus; L, left; R, right; MNI, Montreal Neurological Institute.*

### DTI Discrepancy between Groups

**FIGURE 2:**
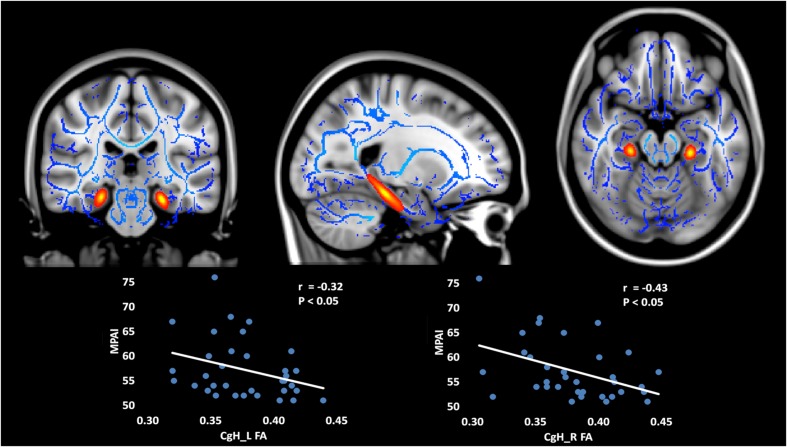
**Decreased FA and AD in bilateral CgH (subregions divided by Johns Hopkins stereotaxic atlases) in MPD group compared with control group (MPD < CG) and the negative correlation between FA and MPAI scores within MPD group.** The FA and AD values of the CgH were significantly decreased in MPD individuals, relative to controls. Furthermore, within the MPD group, the FA of the CgH was negatively correlated with MPAI scores. CgH, cingulum bundle fibers in the hippocampus. FA, fractional anisotropy; AD, axial diffusivity; MPD, mobile phone dependency; CG, control group; MPAI, Mobile Phone Addiction Index.

For the atlas TBSS analysis, the FA and AD values for hippocampal cingulum bundle fibers (CgH) were significantly decreased in MPD individuals, relative to controls (see **Table 3**). Furthermore, within the MPD group, FA of the CgH was negatively correlated with MPAI scores (see **Figure 2**).

**Table 3:** Significant diffusion tensor imaging (DTI) index differences between MPD group and control group (MPD group < control group).

Index	Region	Central MNI Coordinates			*f*	*p*	η*^2^*
		*X*	*Y*	*Z*			
FA	CgH_L	-22	-27	-18	5.98	<0.05	0.08
	CgH_R	24	-25	-19	7.76	<0.01	0.11
AD	CgH_L	-22	-27	-18	6.19	<0.05	0.09
	CgH_R	24	-25	-19	8.01	<0.01	0.11

*CgH, cingulum bundle fibers in the hippocampus; FA, fractional anisotropy; AD, axial diffusivity; L, left; R, right; MNI, Montreal Neurological Institute*.

## Discussion

As far as we know, the present study was the first attempt to explore altered neural morphology in college students with MPD. We compared GMV and four indices of WM integrity (FA, MD, AD, and RD) between MPD individuals and healthy controls. The results revealed that MPD individuals had decreased GMV, relative to controls, in the right sFG, right iFG, bilateral mFG, right mOG, left ACC, and bilateral thalamus (Thal). As far as WM integrity, the MPD group showed decreased FA and AD of bilateral hippocampal cingulum bundle fibers (CgH). Furthermore, GMV values of the right sFG, right iFG, and bilateral thalamus (Thal) were negatively correlated with MPAI scores in the MPD group, as were FA values of the CgH. Additionally, we confirmed that the MPD group had higher trait impulsivity as measured with the Barratt Impulsiveness Scale (BIS-11) (Patton et al., 1995).

These findings indicate possible underlying neurobiological mechanisms behind MPD, and simultaneously help in better understanding the underlying role of impulsivity in this kind of behavioral addiction disorder. The results also provide insight into the similarities and differences between MPD and other kinds of addiction disorders.

### Decreased Gray Matter Volume in MPD

In the present study, we found decreased GMV in the MPD group, a finding that is consistent with our hypothesis based on findings from other drug and behavioral addiction studies where impulsivity is also hypothesized to play an influential role.

Functional neuroimaging studies of drug addiction reveal that drugs of abuse not only influence dopamine-rich subcortical structures such as the ventral tegmental area (VTA), nucleus accumbens (NAcc), caudate nucleus, putamen, thalamus and amygdala, but also disrupt cortical projection areas such as the prefrontal cortex (PFC), OFC, ACC and insula (Hanlon and Canterberry, 2012). Similarly to drug addiction, individuals with behavioral addiction are often characterized as having abnormal function in brain regions that include the prefrontal cortex, ACC (Grant et al., 2010), ventral striatal (Han et al., 2012a) and VTAs, NAcc (Grant et al., 2010), insula (Kuss and Griffiths, 2012) and thalamus (van Holst et al., 2010). Besides altered function in these areas, it is worth noting that altered brain morphology in these areas has also been reported in Internet addicts as well as gambling addicts. For example, studies have shown decreased GMV in these conditions in left ACC, left posterior cingulate cortex, left insula, left lingual gyrus (Zhou et al., 2011), right OFC, bilateral insula, and right supplementary motor area (Weng et al., 2013). These areas largely overlap with circuits related to inhibitory control (Ersche et al., 2011), reward processing, decision-making, and other cognitive functions (Romero et al., 2010).

In our study, decreased volume in three ROIs in the MPD group relative to controls was also correlated with the MPAI. That is, the sFG, right iFG, and thalamus. The sFG has been shown to be involved in many advanced cognitive functions such as inhibitory control, conscious decision-making, reasoning, working memory (Chase et al., 2011), aspects of voluntary top–down attentional control (Hopfinger et al., 2000), and modeling and prediction of the behavior of others (i.e., theory of mind) (Cui et al., 2012).

The right iFG is thought to serve as the control center for fronto-basal ganglia circuits involved with attention distribution, emotional processing, inhibitory control, and behavior monitoring and modulation (Moreno-López et al., 2012). Its injury is associated with behavioral disinhibition, and has been shown to play a key role in the maintenance and exacerbation of behavioral addiction (Grant et al., 2010; Kuss and Griffiths, 2012).

The thalamus, which extensively connects cortical and subcortical structures with one another, could arguably be one of the most crucial hubs of the brain, and has been shown to be related to reward expectation, attention, emotion, memory (Minagar et al., 2013), and executive function (Tuchscherer et al., 2010). Furthermore, thalamic atrophy has been previously found to be associated with cognitive impairment (Hanlon and Canterberry, 2012).

Contrary to our results, a study of patients with online game addiction showed that thalamic volume was actually increased in the addiction group, hypothesized to be the result of higher dopamine availability from the tremendous visual and auditory stimulation that is present in online game playing – altering the balance of mesolimbic circuits (Han et al., 2012b). This contradiction may also represent the difference in acquired habitual behaviors between dependent phone users and computer game-addicted players.

### Abnormal White Matter Integrity in MPD

Besides decreased GMV, we found decreased FA and AD of hippocampal cingulum bundle fibers (CgH) in the MPD group.

The cingulum WM tract carries information from the cingulate gyrus to the hippocampus, and can be separated into two subregions at the axial level of the splenium of the corpus callosum: the cingulum in the cingulate gyrus above the splenium, and the cingulum in the hippocampal regions (CgH) below the splenium (Mori et al., 2008). The function of the CgH involves receiving different combinations of sensory, cognitive, and emotion regulation information. It provides major poly-sensory input to the hippocampus (Zhu et al., 2011), and contributes to reward pathways, and formation, maintenance and retrieval of working memory (Yuan et al., 2011) – information that is critical for cognitive control (Luck et al., 2010; Benedict et al., 2013). Furthermore, abnormal FA in the MPD group is consistent with findings in alcohol addiction (Yeh et al., 2009). Overall, decreased information transmission between the cingulate gyrus and hippocampus, as is suggested by the decreased CgH FA value in the MPD group, may be an underlying structural basis for functional deficits that leads to a solidification of addiction-related memories.

However, as far as we know, decreased FA in the CgH has not yet been reported in any behavioral addiction study. Our data indicates that the reduction of FA in the right CgH of the MPD group was mainly driven by a decrease in AD value, without divergence observed in the RD index. AD measures the magnitude of diffusivity along the principal direction of diffusion, which may index the organization of fiber structure and axonal integrity (Qiu et al., 2008). Thus, the main underlying mechanism of the WM injury in this area present in MPD may be due to subtle axonal injury rather than demyelination (Romero et al., 2010).

### Impulsivity and Mobile Phone Dependence

Impulsivity can be defined as taking actions that are expressed prematurely, that are unduly risky, poorly conceived, and that could result in undesirable consequences (Bickel et al., 2012). It is closely related to a majority of addiction disorders (Romero et al., 2010), such as drug addiction (Moreno-López et al., 2012), pathological gambling (Joutsa et al., 2011), online game addiction (Han et al., 2012b), internet addiction (Lin et al., 2012) and MPD (Billieux, 2012). Interestingly, Walther et al. (2012) investigated the relationship between 12 different personality characteristics and five addictive behaviors including alcohol, tobacco, and cannabis substance use, problematic gambling, and problematic computer gaming, and found that high impulsivity was the only personality characteristic that was associated with all of the addictive behaviors that were investigated.

As expected, we found higher impulsivity in MPD individuals, consistent with previous research. Impulsivity may in fact be the strongest predictor of MPD (Billieux et al., 2008). It can predispose someone to spend extended periods of time on their phone, eventually leading to MPD, and in turn, can further deteriorate self-adaptation and self-control abilities (Billieux et al., 2007). Furthermore, high levels of trait impulsivity are associated with lower midbrain dopamine auto-receptor binding (Buckholtz et al., 2010). As for substance addicted individuals, continued use of stimulants, for example, is thought to further exacerbate impulsive traits (Moreno-López et al., 2012). Thus, because of the close link between impulsivity and addiction disorders, in order to improve treatment interventions that help promote more conscientious and less compulsive decision-making processes, and improve self-control abilities, therapists may focus on reducing traits related to impulsivity (Leung, 2008).

Nevertheless, due to the close relationship between impulsivity and MPD, the difference in BIS scores between the MPD and CG may have been a potential confounding variable in the comparative analysis of mobile phone use. Thus, in our study, BIS score was eliminated as a nuisance regressor in the GMV and FA group contrasts, and values from the resultant regions were then entered into correlation analyses with MPAI scores within the MPD group.

### Limitations and Future Directions

Despite the novel results of the study, there are several limitations to be acknowledged. Firstly, due to the cross-sectional design of the study, and that potential psychological mechanisms are manifold, we cannot infer causality or direction of effect between mobile phone overuse and maladaptive structural alterations found in the MPD group. Thus, longitudinal studies would be very helpful in confirming possible psychological and physiological mechanisms of MPD, as well as provide the ability to measure the length and progression of MPD in participants. Secondly, though we found brain structural differences between the MPD and CG, we cannot know for sure what specific kinds of cognitive function deficits are related to these differences. Future studies should try to connect GMV (or WM integrity) differences in MPD to some psychological function (e.g., cognitive executive function) in order to better inform possible preventive measures and interventions for mobile phone overuse and dependence. Last but not least, despite regressing out BIS scores when performing group difference tests of GMV and FA, the underlying trait impulsivity found in the sample is inevitably an inextricable variable that cannot be completely isolated in the present study. Future studies that perhaps include a comparison between MPD individuals with high impulsivity vs. high-impulsivity non-MPD individuals may be helpful in dissociating MPD from trait impulsivity.

## Author Contributions

YW is responsible for the original experimental design, task procedure, data analysis and article writing. ZZ is responsible for the experimental process, data collection, and article writing. HS is responsible for experiment implementation and data analysis. XX is responsible for behavioral questionnaire data and experimental procedure plan. HW is responsible for chart and graph arrangement, including **Figures 1** and **2**, arrangement of data, and proofreading of the manuscript. FdU is responsible for manuscript writing, description of the functions of brain regions, copy editing, and content editing, as well as approval of the final version to be published. XH is responsible for experimental design and guidance throughout.

## Conflict of Interest Statement

The authors declare that the research was conducted in the absence of any commercial or financial relationships that could be construed as a potential conflict of interest.

## Footnotes

1 http://www.fil.ion.ucl.ac.uk/spm

2 http://www.rfmri.org/dpabi

3 http://www.restfmri.net

4 http://www.nitrc.org/projects/panda/

5 http://www.fmrib.ox.ac.uk/fsl/tbss
